# Applicability of Complementary Colors in Skin Tone Correction for Young Chinese Adults Based on Image Processing and Machine Learning

**DOI:** 10.1111/jocd.70566

**Published:** 2025-11-24

**Authors:** Guolong Dong, Yueheng Liu, Jianghong Ran, Fan Yi, Li Li, Hong Meng, Yue Wu

**Affiliations:** ^1^ Beijing Key Laboratory of Plant Resources Research and Development Beijing Technology and Business University Beijing China; ^2^ Key Laboratory of Cosmetic, China National Light Industry Beijing Technology and Business University Beijing China; ^3^ Institute of Cosmetic Regulatory Science Beijing Technology and Business University Beijing China; ^4^ Beijing Academy of TCM Beauty Supplements Co. Ltd Beijing China

**Keywords:** Chinese population, colorimetric analysis, complementary colors, skin tone correction

## Abstract

**Background and Objective:**

Skin tone correction is an essential focus within dermatology and cosmetology, particularly in achieving a balanced and even facial appearance. The application of complementary color theory in skin tone correction remains predominantly subjective, relying on individual user experiences rather than systematic and quantitative assessments. This study aims to evaluate the applicability of complementary color theory among young Chinese individuals and develop predictive models for personalized skin tone correction.

**Methods:**

Sixteen young Chinese female participants aged 20–25 were recruited. Standardized facial images were captured using the VISIA‐CR system under standardized lighting conditions, both before and after the application of six color‐correcting primers (orange, pink, blue, white, purple, and green). Four facial regions of interest (ROIs), defined as the forehead, under‐eye circles, cheeks, and near‐nose, were analyzed. Five colorimetric indices (L*, a*, b*, ITA°, and Hab°) were quantified across each ROI. State‐of‐the‐art machine learning regression models were developed to predict post‐application ITA° and Hab° values based on pre‐application skin tone and primer characteristics.

**Results:**

Under‐eye circles exhibited the darkest and most yellowish‐red skin tone compared to other regions. Complementary color primers demonstrated statistically significant improvements in ITA° and Hab° values across all ROIs. Pink primers were most effective for under‐eye dark circles, while purple, pink, and blue primers resulted in greater improvements in overall skin tone. LightGBM and XGBoost regression models demonstrated superior performance, with *R* ^2^ values reaching 0.824 for ITA° and 0.850 for Hab°.

**Conclusion:**

This study robustly validates the efficacy of complementary color primers in skin tone correction among young Chinese individuals. The integration of machine learning offers a robust framework for personalized cosmetic recommendations, paving the way for innovative and data‐driven advancements in skincare and makeup applications.

## 1 Introduction

A balanced and uniform facial appearance is recognized as a crucial determinant in assessing human attractiveness, health, youthfulness, and vitality [1, 2, 3, 4, 5, 6]. However, achieving this balance is often hindered by factors such as discoloration, redness, or hyperpigmentation, leading to uneven skin tones. Skin tone correction, also referred to as skin camouflage, has gained increasing attention in dermatology and cosmetology. Complementary color theory serves as a foundational principle in the development of color‐correcting makeup products, where opposite hues on the color wheel neutralize specific imperfections.

In colorimetry, complementary color stimuli are defined as pairs of colors that, when combined in appropriate proportions, match an agreed achromatic color stimulus, such as a particular CIE illuminant. Two colors are considered complementary only if they exhibit the correct relative luminances (or alternatively, relative radiant powers) and chromaticities to precisely produce a neutral additive mixture matching the agreed achromatic stimulus [7]. Complementary colors exhibit striking visual contrast, characterized by enhanced vividness and purity when juxtaposed. For example, when red and green hues are placed side by side, their interaction increases their perceived intensity, creating a vivid visual contrast that captures attention and evokes specific psychological effects.

Corrective cosmetics utilizing complementary color theory play a pivotal role in dermatology and cosmetology. These products are applied to address various conditions that affect skin color, such as hemangiomas, birthmarks, vitiligo, lupus erythematosus, port wine stains, postoperative sequelae, scars, and leukoderma [8]. Such cosmetics not only conceal imperfections but also provide emotional benefits for individuals with compromised skin. For instance, studies have reported a significant 30% improvement in Skindex‐16 scores after 3 months of corrective cosmetic use in patients with disfiguring facial disorders [8]. Additionally, products with a slight green tint have been found effective in camouflaging redness in individuals with rosacea [9], while others improve oil control, offer sun protection, and enhance personal well‐being [10].

Chinese individuals exhibit distinct and unique facial skin color characteristics. According to our previous research, the primary skin tone categories of Chinese females range from light (I) to tan (IV), based on Individual Typology Angle (ITA°) grades, with varying levels of redness and yellow pigmentation [11]. Significant anatomical differences in skin tone have also been observed, such as higher ITA values in the forehead and lower values in the jaw [12]. These features present specific challenges in skin tone correction, as redness and dullness are often more pronounced compared to other populations. Environmental factors, such as UV exposure and pollution, exacerbate pigmentation issues, further emphasizing the need for effective and personalized correction methods.

The Hue Angle (Hab°) is essential in skin tone analysis as it directly measures color variations, such as redness or yellowness, in the skin [12]. It is crucial for skin tone correction in cosmetics, allowing for precise adjustments and better matching of undertones. Hab° provides a clearer representation of color shifts, making it important for accurate prediction models and personalized skincare treatments. By integrating Hab° with other metrics, it enhances the overall effectiveness of skin tone correction and cosmetic formulations.

Image Processing has enabled the development of automated systems capable of quantifying skin parameters with high precision, overcoming the limitations of subjective human judgment [13, 14]. On the other hand, recent advances in machine learning (ML)‐based predictive modeling have revolutionized predictive tasks by enabling data‐driven, personalized recommendations. XGBoost (eXtreme Gradient Boosting) and LightGBM (Light Gradient Boosting Machine), which are based on Gradient Boosting Decision Tree (GBDT), are both popular and cutting‐edge boosting integrated algorithms in machine learning in recent years. XGBoost is a scalable and regularized boosting framework that uses second‐order Taylor approximation to optimize the loss function, enabling robust performance and effective handling of sparse data [15]. An AI‐based patient selection tool prior to cosmetic surgery using the XGBoost algorithm has been developed with a high level of agreement with surgeons' evaluation [16]. LightGBM is a gradient boosting framework that introduces histogram‐based decision tree learning and a leaf‐wise growth strategy, significantly accelerating training speed and reducing memory usage [17]. Yang H. et al. [18] improved the LightGBM model to predict coronary heart disease with an AUC value of 97.8%, better than other models. Both of them have the virtue of less training time and higher accuracy.

**FIGURE 1:**
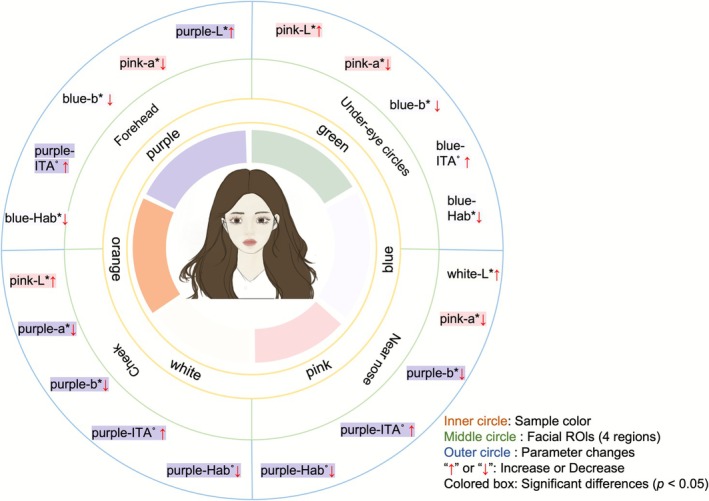
Graphical summary.

Despite its popularity, the application of color theory in skin tone correction remains largely subjective, relying on individual user experiences rather than systematic and quantitative evaluation. Research on colorimetric analysis of makeup effects in East Asian populations, particularly young Chinese adults, remains relatively underexplored. To address this gap, this study integrates state‐of‐the‐art image processing and machine learning techniques to quantitatively assess the effectiveness of complementary color primers. By analyzing the effects of six color primers (orange, pink, blue, white, purple, and green) across four specific anatomical sites using objective colorimetric indices, this research aims to establish a scientific foundation for personalized skin tone correction and advance the understanding of product performance in this demographic (Figure 1).

## 2 Methods and Materials

### 2.1 Study Design

This study enrolled 16 healthy Chinese female volunteers aged 20–25 years. Participants meeting any of the following exclusion criteria were excluded: (a) presence of skin diseases, (b) pregnancy, (c) sensitivity to sunlight, (d) administration of immunosuppressive drugs within the past 3 months, (e) receipt of any form of phototherapy within the past 6 months, and (f) visible skin lesions at the measurement sites. The study was strictly conducted according to the principles of the Declaration of Helsinki. Informed consent was obtained from all participants after providing a thorough explanation of the study protocol.

**FIGURE 2:**
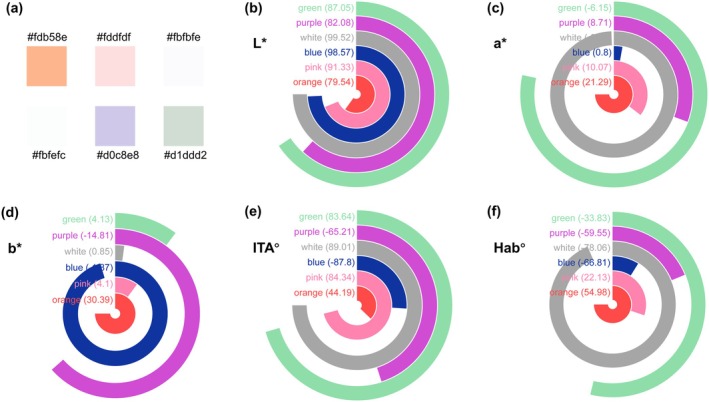
Sample color index: (a) sample color; (b) sample difference in L* value; (c) sample difference in a* value; (d) sample difference in b* value; (e) sample difference in ITA° value; (f) sample difference in Hab° value.

All volunteers sequentially applied six color‐correcting primers to the whole facial area. To ensure accurate and reliable data, volunteers are required to thoroughly clean their facial skin, wait for 20 min and use an equal amount of primer every time to avoid any skin reactions that may be triggered by cleansing products, which could interfere with the experimental outcomes. Facial images were captured using the VISIA‐CR system under standardized lighting conditions both before and after applying six color‐correcting primers: orange, pink, blue, white, purple, and green (Figure 2). Participants adhered to a standardized cleansing protocol prior to image acquisition.

### 2.2 Image Processing

**FIGURE 3:**
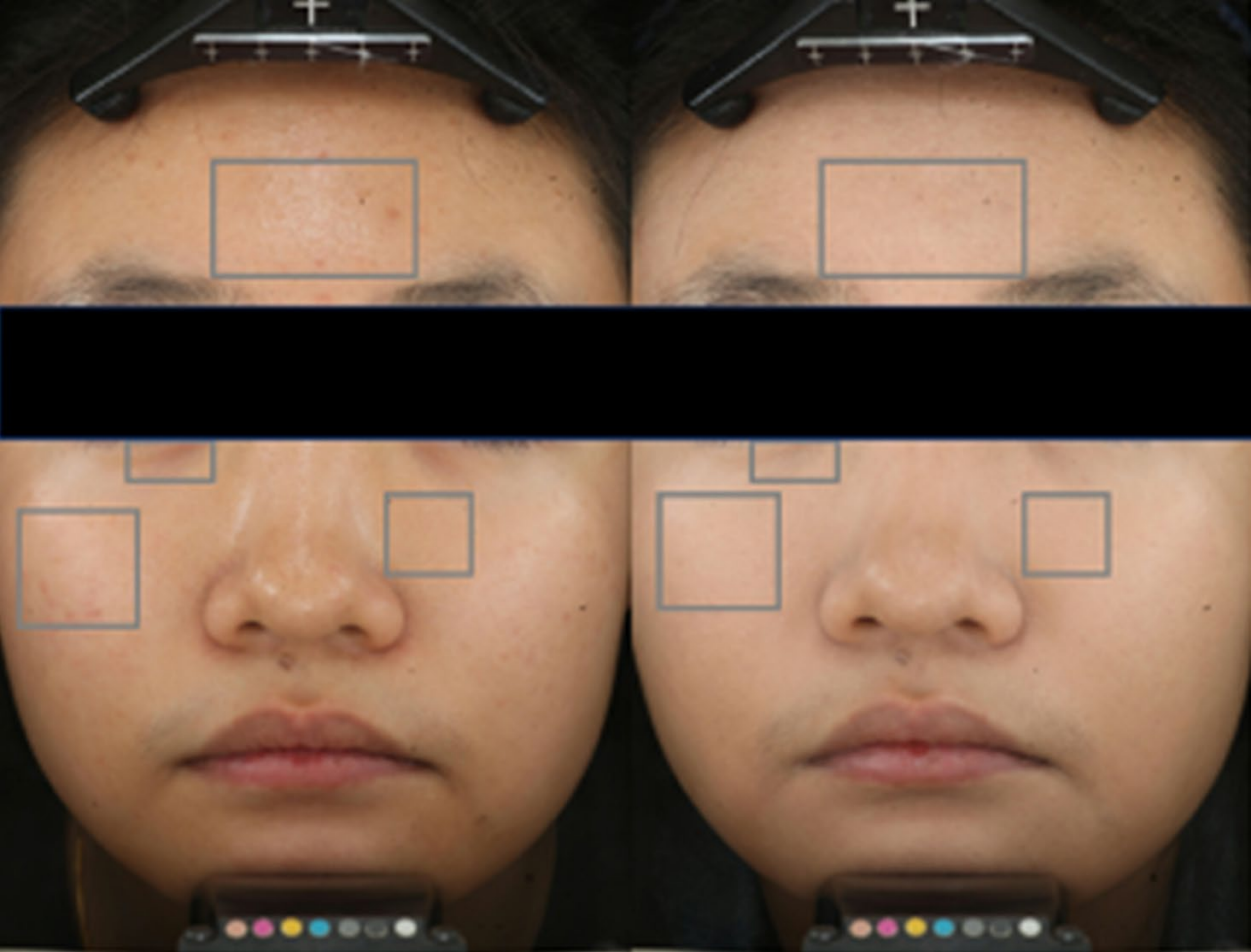
ROI of facial anatomical sites (before and after primer application). Four areas are the forehead, under‐eye circles, near‐nose and cheek.

Facial images were captured using the VISIA‐CR system with a standardized lighting source. Regions of interest (ROIs) were precisely extracted from four facial areas: the forehead, under‐eye circles, cheeks, and near the nose (Figure 3).

Skin color parameters were analyzed using the CIE‐L*a*b* color model, which aligns with human visual perception and color understanding. Five parameters were extracted: L* (lightness), a* (redness), b* (yellowness), Individual Typology Angle (ITA°), and Hue Angle (Hab°). ITA°, introduced by Chardon in 1991, quantifies skin pigmentation as the angular direction in the L*‐b* plane and is calculated as:

(1) $${\mathrm{ITA}}^{{}^{\circ}}=\tan^{-1}\left(\frac{L*-50}{b*}\right)\times \frac{180}{\pi }$$

Skin color is classified into six categories based on ITA° values, inversely proportional to skin pigmentation levels. Similarly, Hab°, derived from the CIE‐L*a*b* color space, quantifies erythema using redness (a*) and yellowness (b*) and is calculated as:

(2) $${\mathrm{Hab}}^{{}^{\circ}}=\tan^{-1}\left(\frac{b*}{a*}\right)\times \frac{180}{\pi }$$

Skin Hab° primarily range from 0° to 90°, with lower values indicating higher erythema, while higher values indicating higher yellowness.

### 2.3 Statistical Analysis

All statistical analyses were conducted using SPSS 26.0 (SPSS Science, Chicago, IL) software. Data were expressed as mean ± standard deviation (SD). Descriptive statistics were used to analyze baseline facial skin tones across different facial regions and the changes before and after primer application. One‐way analysis of variance (ANOVA) was used to evaluate differences in skin tones across facial regions. Two‐way ANOVA was used to assess interaction effects between facial regions and primer colors on post‐application skin tone indicators. For the two‐way ANOVA, post hoc analyses were conducted using the Least Significant Difference (LSD) test to explore specific group differences. A significance level of *p* < 0.05 was considered statistically significant.

Pearson correlation analysis was employed to explore relationships among skin tone metrics before and after primer application. Additionally, two machine learning regression algorithms, XGBoost and LightGBM, were employed to construct predictive models for post‐application skin tone based on pre‐application skin tone and primer color attributes. Input features included L*, a*, b*, sample‐*L*, sample‐a*, and sample‐b*. Output features are Post‐makeup ITA° and Hab° values. The dataset was randomly shuffled and split into training (80%) and testing (20%) subsets, and 3‐fold cross‐validation was applied during model training. Model evaluation was based on standard regression metrics, including MSE, RMSE, MAE, MAPE, and *R* ^2^. All model parameters were optimized using default settings with appropriate regularization to ensure generalizability.

## 3 Results

### 3.1 Facial Skin Tone Status in Different Anatomical Sites

**FIGURE 4:**
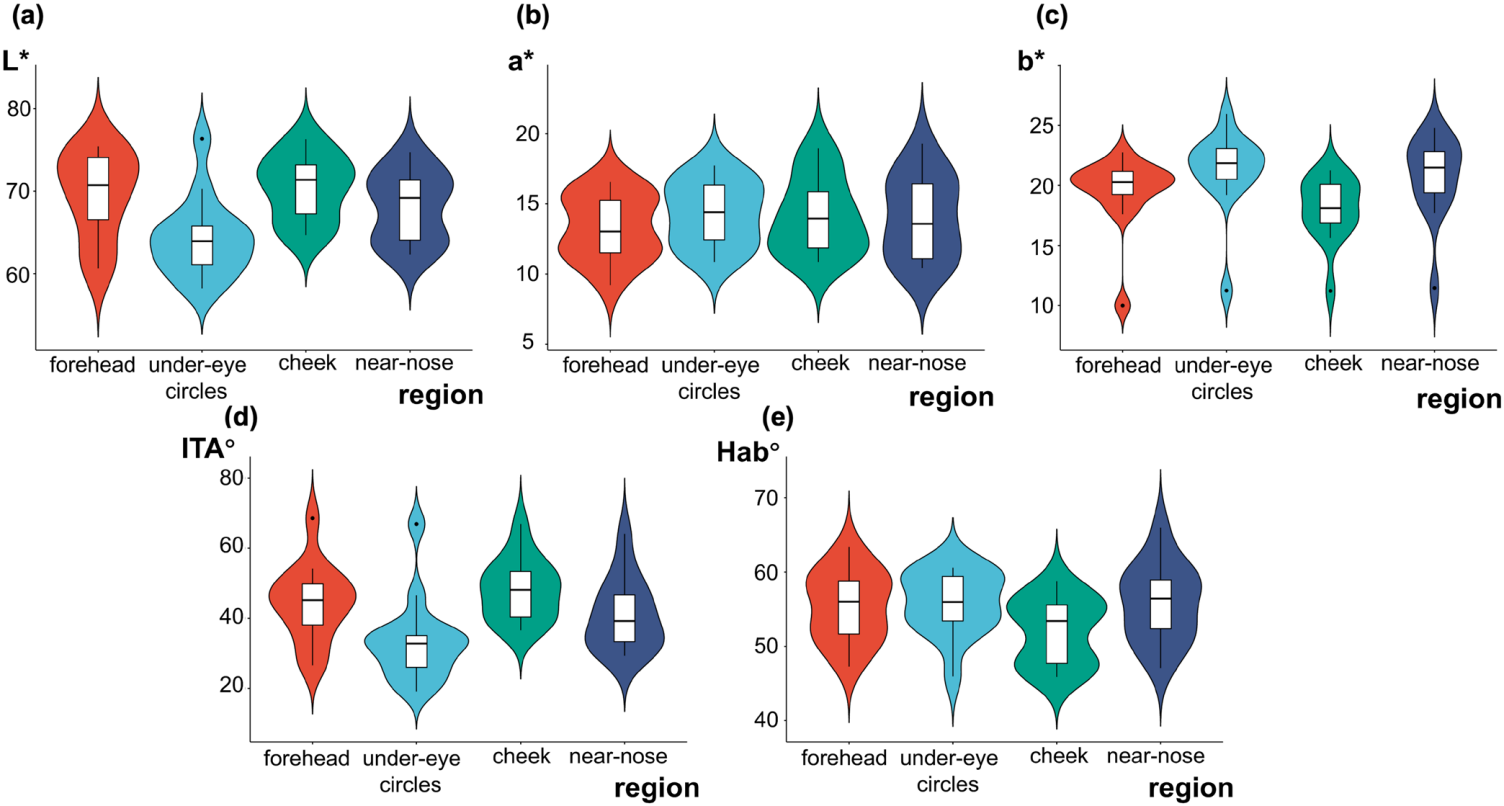
Color difference among the region of facial skin: (a) L* value in four facial regions; (b) a* value in four facial regions; (c) b* value in four facial regions; (d) ITA° value in four facial regions; (e) Hab° value in four facial regions.

The analysis of facial skin tone across various anatomical regions reveals notable differences in skin tone indexes, as summarized in Figure 4 and Table S1. The bars within Figure 4 represent the interquartile range (IQR), and the black central line within the box represents the median value. Additionally, the width of the violin plots reflects the distribution density of the data.

The results revealed that the cheek area exhibited the highest lightness index (L*), with a mean value of 70.43, significantly surpassing that of the under‐eye circles. This indicates a brighter tone, which aligns with perceptions of healthy, youthful skin. In contrast, the under‐eye circles displayed the lowest mean lightness index at 64.26, reflecting a darker tone commonly associated with fatigue or the aging process.

On the red‐green axis (a*), the forehead and cheek areas displayed similar values, indicating moderate redness, while the under‐eye circles and near‐nose regions showed slightly higher values, potentially linked to vascularity or localized conditions.

In terms of yellowness (b*), the forehead recorded the lowest value with 19.51, while the under‐eye circles reflected higher yellowness, which may contribute to a perceived dullness in this region. ITA° values further substantiated the findings, with the forehead and cheeks exhibiting lighter skin types, whereas the under‐eye circles leaned toward darker classifications.

Hab° values remained relatively consistent across regions, which proved Chinese had specific skin color features and suggested localized differences due to pigmentation and vascular factors.

### 3.2 Description of Color Cosmetics and Complementary Color

**FIGURE 5:**
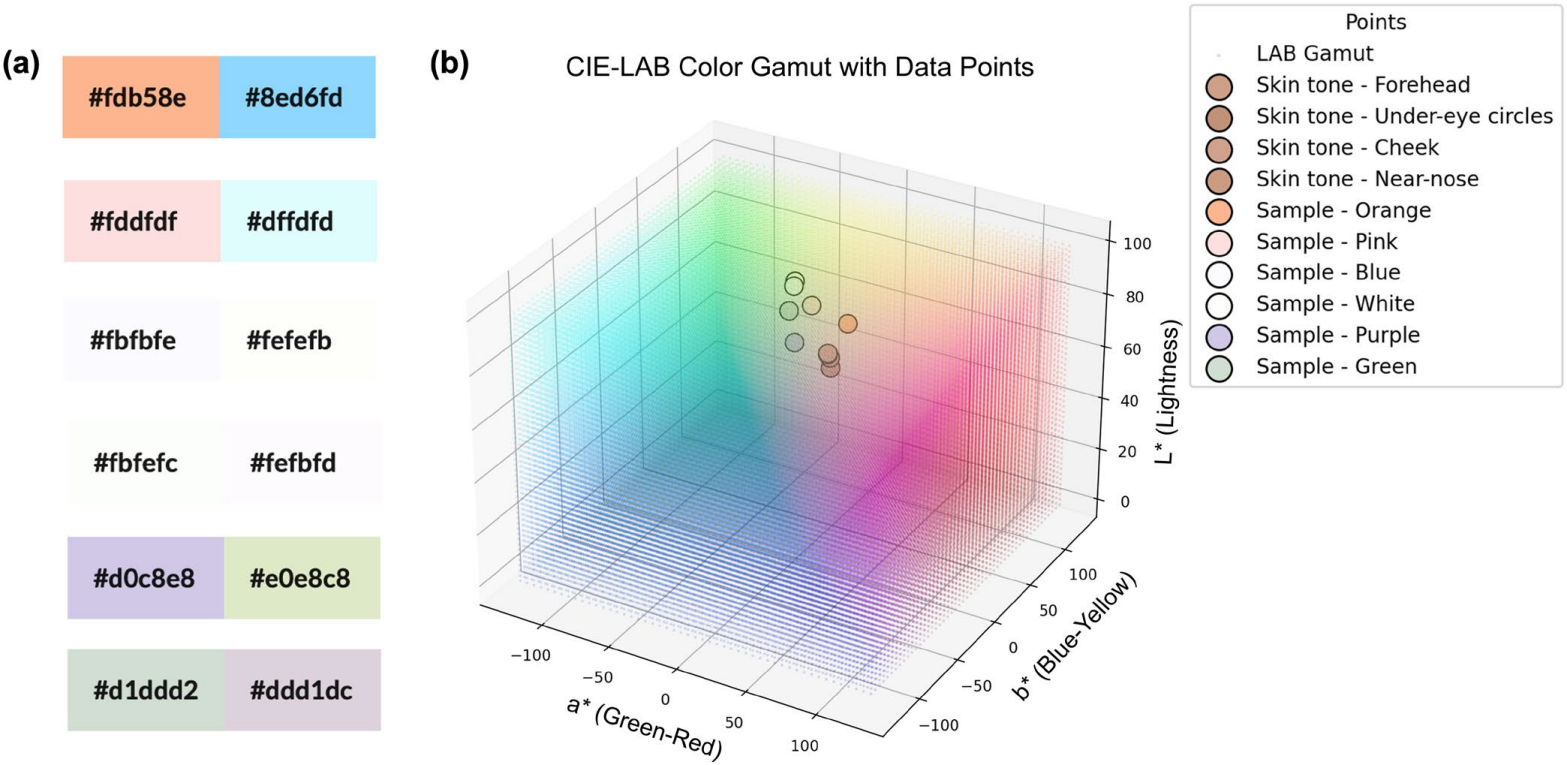
Complementary color: (a) primer color and correlated complementary color; (b) spatial distribution of skin tone and sample color in CIE‐L*a*b* color space.

The color metrics of color cosmetics is shown in Figure 2a–f and their complementary colors are illustrated in Figure 5a, which indicated that orange, with the lowest L* value of 79.54, exhibited a blend of red and yellow undertones, making it suitable for correcting skin tones with excess blue hues. Pink, characterized by the lightness (L* = 91.33) and minimal chromaticity (a* = 10.07, b* = 4.10), served effectively to brighten and neutralize light blue undertones in the skin.

Blue displayed an exceptionally high L* value of 98.57 but a negative b* value (−1.87), which suggested its effectiveness in counteracting warm undertones, particularly beneficial for individuals with warmer complexions. On the other hand, white presented a versatile option in highlighting features while providing overall correction with the highest lightness.

Purple demonstrated a moderate L* value with a strongly negative b* value, making it suitable for neutralizing yellow‐green undertones, resulting in a balanced complexion. Green, with L* = 87.05 and a negative a* value, was effective in reducing red tones, beneficial for conditions like rosacea.

Quantitative analysis of the CIE‐L*a*b* color space revealed a strategic distribution between skin tones and corrective colors. While the skin tones clustered in the yellow‐red region, the corrective samples were located in the opposing blue‐purple quadrant, confirming a near‐complementary relationship for hue neutralization. More importantly, as visualized in Figure 5b, all samples were positioned in the upper‐left direction relative to the skin tones. This specific spatial distribution indicates that the color correction strategy achieves a dual purpose: neutralizing unwanted undertones through complementary hues while simultaneously increasing skin lightness (L*) and reducing chroma (a* and b*). In cosmetic applications, the core principle of using complementary colors extends beyond mere hue cancellation; it must also integrate adjustments in luminance and chroma to achieve a balanced, brightened, and natural‐looking complexion.

### 3.3 Comparison of Facial Skin Tone Before and After Makeup Application

**FIGURE 6:**
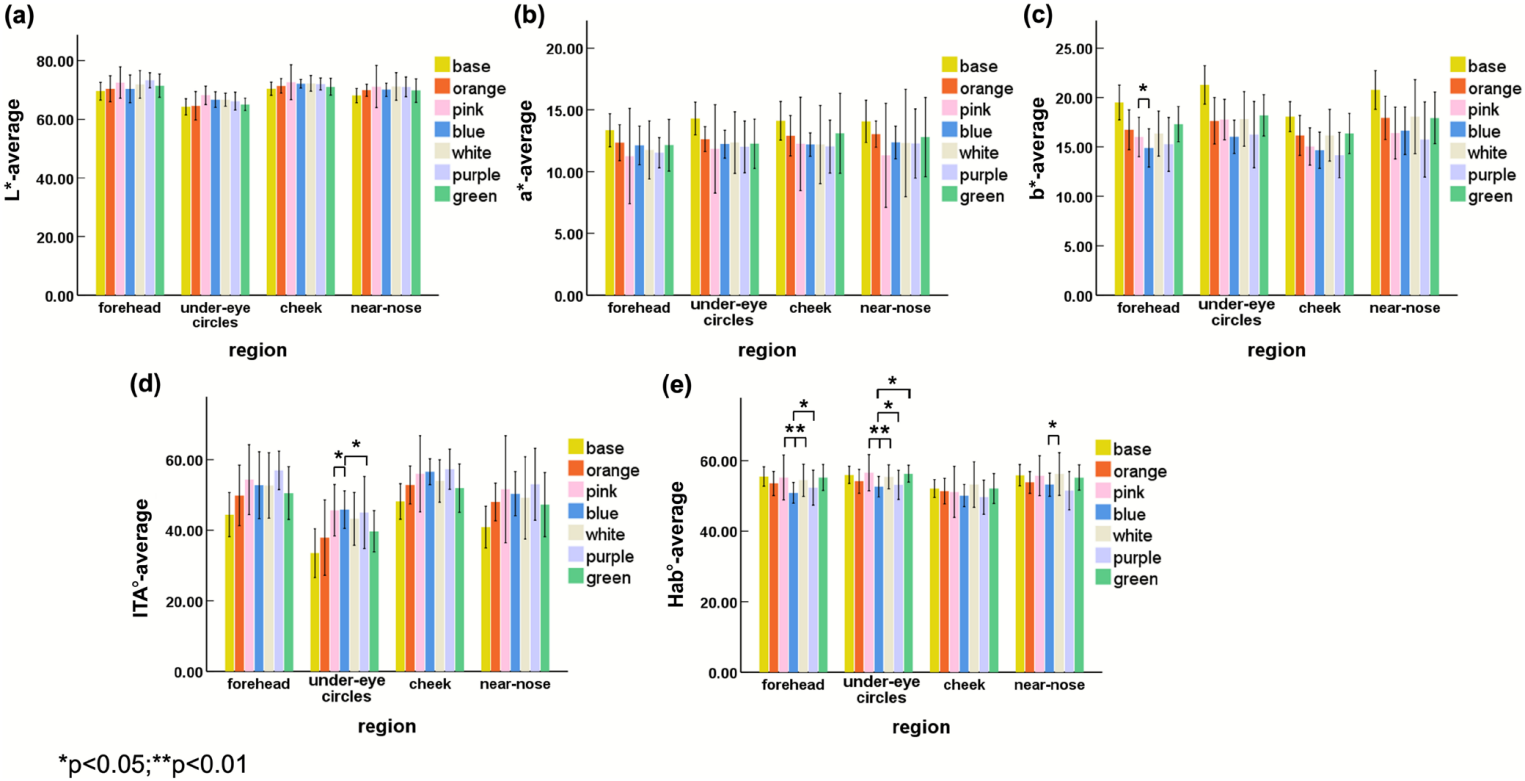
Distribution of descriptive and differential analysis for the skin tone indexes measured across different sample colors and anatomical regions：(a) L* value in four facial regions; (b) a* value in four facial regions; (c) b* value in four facial regions; (d) lTA° value in four facial regions; (e) Hab° value in four facial regions. (“base” indicates skin tone before using makeup application; “other colors” indicate skin tone after using makeup application).

The comparison of facial skin tone before and after makeup application is summarized in Figure 6 and Table S2. Descriptive and differential analyses were conducted for skin tone indices across sample colors and anatomical regions.

Across different regions of the face, the mean values for the parameter L* ranged from 64.26 to 73.32, with the highest mean observed in the forehead region under the purple sample color. The parameter a* exhibited mean values between 11.26 and 14.31, with the forehead region in the pink sample color displaying the lowest mean value. Meanwhile, the mean values for b* ranged from 14.89 to 21.29, with the under‐eye circles in the base color showing the highest mean. The ITA° parameter varied significantly, with mean values from 33.47 to 57.25, while the Hab° readings ranged from 49.58 to 56.56, indicating variability across sample colors and facial regions.

**FIGURE 7:**
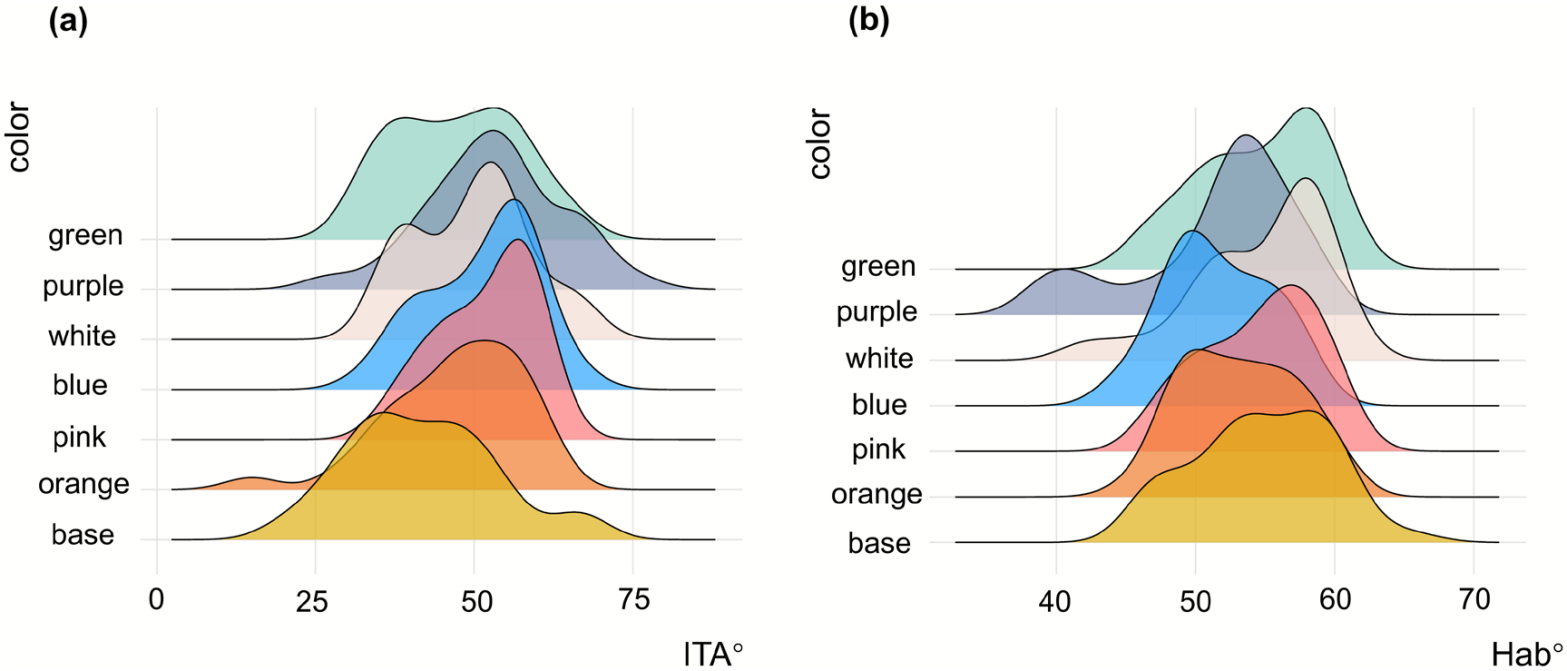
Comparison of color index among different color primers: (a) ITA° difference; (b) Hab° difference.

The results indicated that the purple color showed pronounced trends toward the desired outcome: it was associated with an increase in L* and ITA°, and a decrease in a*, b*, and Hab°, particularly in the cheek region. The pink color exhibited a trend in reducing a* values on the forehead, under‐eye, and near‐nose regions. Similarly, the blue color showed the trend in decreasing b* values on the forehead and under‐eye areas. These observed trends are consistent with color theory, as purple (complementary to yellow) and blue are theorized to effectively neutralize yellow undertones (reducing b* and increasing perceived lightness L*), while pink can add radiance, even though the differences in mean values between samples were not statistically significant. Regardless of regions, it showed that all colors improved similar trends in ITA° with a lighter and brighter direction (Figure 7a), while it showed a different direction on Hab° values (Figure 7b).

A two‐way ANOVA was conducted to analyze the effects of sample color and facial anatomical sites on five color indexes, including L*, a*, b*, ITA°, and Hab°. The results indicated that there were three indexes with significant main effects for both factors, which are b*, ITA°, and Hab°, as shown in Table 1. Only the region had a significant main effect on L* with the effect of region being significant, and the effect of Group also being significant. Although the interaction effect between Group and Region was not significant on all color indexes, post hoc analysis revealed that the L* scores for the cheeks were significantly higher than those for under‐eye circles and near‐nose. b* scores for the cheeks were significantly lower than those for under‐eye circles and near‐nose. Under‐eye circles had the lowest ITA° among four regions with a significant difference (*p* < 0.01). Hab° scores for the cheeks were significantly lower than those of other facial areas.

**TABLE 1:** Two‐way ANOVA analysis result.

Index	Term	Sum of squares	Degrees of freedom	Mean square	*F*	*p*
L*	Intercept	771 745.337	1	771 745.337	53 947.865	0.000***
	Group	81.371	5	16.274	1.138	0.343
	Region	819.803	3	273.268	19.102	0.000***
	Group region	44.024	15	2.935	0.205	0.999
a*	Intercept	23 454.341	1	23 454.341	3912.712	0.000***
	Region	7.905	3	2.635	0.44	0.725
	Group	18.89	5	3.778	0.63	0.677
	Region group	5.392	15	0.359	0.06	1.000
b*	Intercept	42 681.655	1	42 681.655	5657.235	0.000***
	Region	91.247	3	30.416	4.031	0.009***
	Group	120.294	5	24.059	3.189	0.009***
	Region group	10.834	15	0.722	0.096	1.000
ITA°	Intercept	393 739.546	1	393 739.546	5109.017	0.000***
	Region	3186.597	3	1062.199	13.783	0.000***
	Group	910.875	5	182.175	2.364	0.043**
	Region group	126.53	15	8.435	0.109	1.000
Hab°	Intercept	448 229.441	1	448 229.441	21 765.775	0.000***
	Region	278.941	3	92.98	4.515	0.005***
	Group	319.015	5	63.803	3.098	0.011**
	Region group	50.288	15	3.353	0.163	1.000

** *p* < 0.01.

*** *p* < 0.001.

Moreover, post hoc analysis results by LSD showed that the blue group had a significantly lower Hab° index compared to the green, pink, and white groups, and there was also a significant difference between the blue and pink groups, indicating that different sample colors significantly affected the Hab° index. Comparisons among different color primers demonstrated that the Hab° index for the cheeks was significantly lower than that for the forehead, under‐eye circles, and near the nose, while there was no significant difference among all color primers on L* value. The blue group had a significantly lower b* value compared to the green, orange, and white groups. The purple group had the lowest b* value compared to other color groups, while there were significant differences with the green and orange groups. Simultaneously, the purple group had the highest ITA° value, and compared to the green and orange groups with significant differences.

### 3.4 Adaptation of Facial Skin Tone Between Before and After Make‐Up Status

**FIGURE 8:**
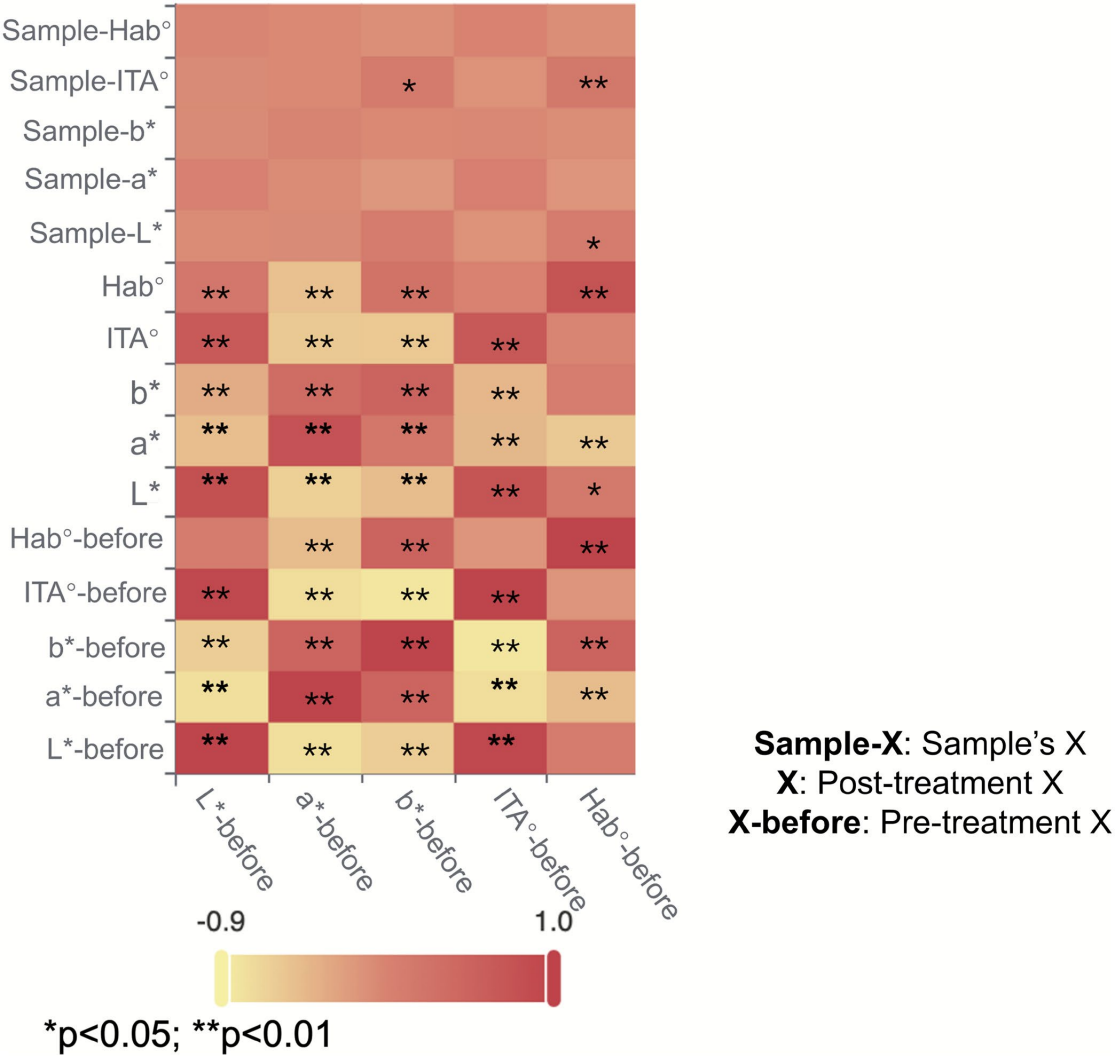
Correlation heatmap.

The Pearson correlation heatmap (Figure 8) reveals significant relationships among various dimensions before and after makeup application. The L*‐before variable exhibits strong negative correlations with both a*‐before (−0.757) and b*‐before (−0.601), suggesting a potential inverse relationship between these parameters. Conversely, L* demonstrates a robust positive correlation with ITA°‐before (0.948), indicating that higher L* values correspond to increased ITA°. The dimensions of a* and b* are positively correlated with each other (0.596) and exhibit significant negative correlations with other factors, such as becoming negatively correlated with L* (−0.467) and ITA° (−0.416). Furthermore, after the intervention, Sample‐a* displays a significant positive correlation with Sample‐b* (0.564) and Sample‐ITA° (0.274). Notably, both b*‐before and Hab°‐before show significant correlations with Sample‐ITA°, which may reveal the adaptation of facial skin tone before and after makeup.

The adaptation of facial skin tone before and after makeup application was further evaluated using advanced machine learning techniques. Post‐makeup ITA° and Hab° were treated as the dependent variables, while pre‐makeup L*, a*, b*, and the corresponding values of the makeup samples' L*, a*, and b* parameters served as independent variables. We tested various regression models and found that LightGBM was the most effective for predicting ITA° due to its ability to handle high‐dimensional data, while XGBoost performed best for predicting Hab° because of its strength in capturing non‐linear relationships in skin hue variations. These model choices were based on extensive cross‐validation and performance evaluation. ITA° regression evaluation results are shown in Table 2. The LightGBM regression model achieved a coefficient of determination (*R* ^2^) of 0.824 for the test set, indicating strong predictive capability in explaining post‐makeup ITA° variance based on pre‐makeup color values.

**TABLE 2:** ITA° regression evaluation results.

	MSE	RMSE	MAE	MAPE	*R* ^2^
Train	4.58	2.14	1.511	3.368	0.954
Cross‐validation	36.227	6.005	4.225	9.179	0.613
Test	11.153	3.34	2.189	4.462	0.824

**FIGURE 9:**
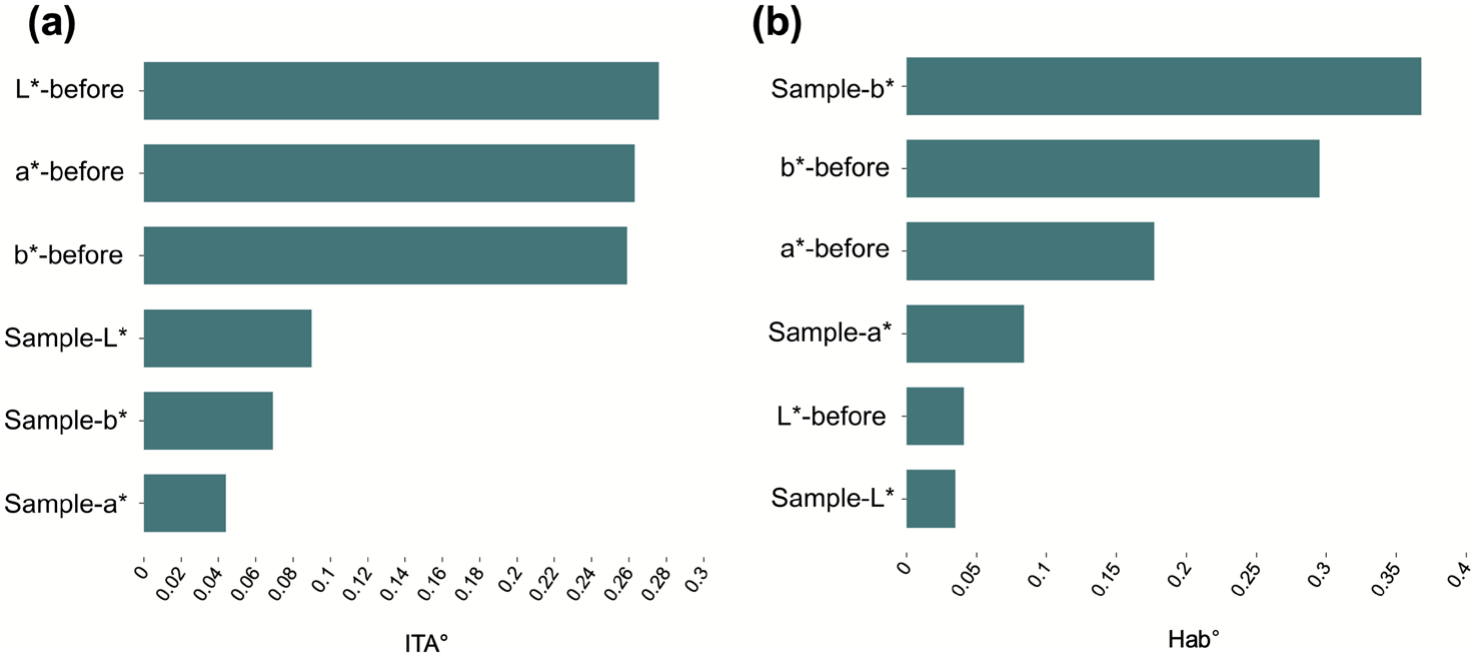
Feature importance between ITA° and Hab°: (a) Feature importance result of ITA°; (b) Feature importance result of Hab°.

Feature importance analysis for the ITA° model revealed the ranked contributions of each feature (Figure 9a): Among all evaluated variables, L*‐before demonstrated the highest contribution to the model, with a*‐before ranking as the second most significant predictor. In contrast, the contributions of the makeup sample features—Sample‐L*, Sample‐a*, and Sample‐b*—were notably lower. These results highlight the dominant role of pre‐makeup color parameters compared to the relatively minor impact of makeup sample color characteristics.

The XGBoost regression model produced a higher *R* ^2^ value of 0.850 for the test set in the Hab° regression model (Table 3), indicating even stronger performance in predicting the effectiveness of makeup in altering facial skin tone yellowness and redness. The feature importance analysis for the Hab° model showed that the sample's yellow‐blue axis (sample‐b*) emerged as the most significant feature (Figure 9b). This suggests that in predicting Hab°, the characteristics of the makeup samples play a more dominant role, particularly the yellow‐blue axis, which significantly influences the overall color change.

**TABLE 3:** Hab° regression evaluation results.

	MSE	RMSE	MAE	MAPE	*R* ^2^
Train	0.004	0.063	0.037	0.07	1
Cross‐validation	7.682	2.766	2.193	4.204	0.628
Test	3.166	1.779	1.317	2.501	0.85

## 4 Discussion

This study conducted a comprehensive evaluation of the suitability of six commonly used complementary color primers across different facial regions in young Chinese individuals and developed two predictive models for personalized skin tone correction based on ITA° and Hab°. As shown in Figure 1, results indicated that the purple primer significantly improved ITA° values in most facial zones, particularly by reducing both redness and yellowness in the cheek area, thereby shifting the skin tone from a yellowish to a more reddish appearance. The blue primer was especially effective in the under‐eye region, demonstrating strong performance in reducing yellowness and enhancing overall skin brightness. In addition, the pink primer showed notable efficacy in increasing skin lightness and diminishing redness, contributing to a more luminous and even complexion.

Elevated melanin accumulation, reduced hemoglobin visibility, thinner periorbital skin, and increased vascular visibility (due to diminished subcutaneous fat) collectively contribute to under‐eye darkness. These anatomical factors, coupled with oxidative stress and poor lymphatic drainage, contribute to the discoloration commonly observed under the eyes [19]. A comprehensive analysis of skin tone across four anatomical sites revealed that under‐eye circles exhibit the lowest lightness and highest darkness values, alongside the most pronounced redness and yellowness, with statistically significant differences. These findings align with the underlying physiological mechanisms. Our results affirmed that pink primers were most effective in correcting under‐eye circles. This is attributable to the higher concentrations of blue pigments in the area, while by the principles of subtractive color mixing, pink, being a red‐leaning complementary to blue‐green hues, neutralizes the bluish cast of under‐eye shadows. As complementary colors, pink neutralizes these pigments, creating a more balanced tone that aligns closely with the surrounding skin. Skin blood perfusion and oxygenation, which are influenced by cardiovascular, hormonal, and circulatory health, play an important role in perceived health and socio‐sexual signaling, as highlighted by Stepen et al. [20] Furthermore, Tan et al. [21] analyzed under‐eye circles in 269 Chinese women and reported significant pigmentation differences in the eyelids, corroborating our findings.

Purple primers, likewise, demonstrated notable efficacy on the cheeks, an area often affected by uneven melanin dispersion and mild erythema [22]. Conversely, while green primers effectively reduced redness, their overall efficacy was not optimal in this study, potentially due to variations in skin type, redness intensity, or primer formulation, or facial skin redness that is highly individualized and influenced by both vascular and pigmentary factors, including inflammation, acne, and capillary fragility [23, 24].

The efficacy of complementary colors in improving skin tone imperfections on specific facial anatomical sites was confirmed among young Chinese adults. Color is a universal perceptual stimulus with significant aesthetic value [25]. When compared to orange, green, and white primers, purple, blue, and pink primers showed better adaptability to the skin tones of young Chinese individuals. Purple pigments, often derived from plant‐based anthocyanins like those found in purple sweet potato, purple corn, and elderberry, have been successfully used as natural colorants in cosmetic formulations [26].

Furthermore, the study integrated image processing and machine learning techniques to offer a novel, data‐driven pipeline for personalized skin tone correction. Unlike traditional approaches that rely on general tone categorizations or user‐reported preferences, our method extracts precise colorimetric features from facial regions and feeds them into optimized machine learning regressors to predict perceptual skin tone metrics (ITA°, Hab°). This quantitative and objective evaluation not only improves prediction accuracy but also enhances usability for both R&D and consumer applications. The identification of L*—before as a key predictor for ITA° in the LightGBM model is consistent with earlier studies indicating that lightness correlates strongly with perceived evenness and luminosity [27, 28]. Conversely, sample‐b* as a predictor of Hab° in the XGBoost model reflects the perceptual weight of yellowness in evaluating skin uniformity [29]. These results suggest that gradient boosting models are well‐suited for capturing complex, nonlinear relationships between facial chromatic data and perceived skin tone metrics. These insights strengthen the rationale for AI‐driven personalized cosmetic recommendations, as demonstrated in prior work by Shi et al. [30] and Lee et al. [31], which emphasize real‐time visual simulations and ingredient‐function prediction using deep learning. AI with images has offered personalized, efficient, and result‐driven outputs in cosmetic dermatology, such as in cosmetic consultations, assessing outcomes, treatment prediction, and patient education [32]. Furthermore, XR (Extended Reality) and AI are being combined to develop immersive skincare experiences. Rajegowda et al. [33] achieved a 93% accuracy rate in personalized cosmetic product recommendations by leveraging CNNs and XR technologies. However, most algorithms focus on the skincare field, while colored cosmetics still need more research on personalized recommendation. The application of complementary color theory extends beyond cosmetic camouflage [34, 35, 36], aligning with diverse personal preferences and enhancing user satisfaction.

This study quantitatively evaluated the application of complementary color cosmetics among young Chinese adults, highlighting their efficacy in correcting skin tone unevenness. By integrating image processing and machine learning, the research presents a robust approach for personalized skincare and cosmetic recommendations. Complementary color theory demonstrated its potential as an effective tool for improving skin tone uniformity and can inspire further development of computer vision algorithms for cosmetic applications.

However, this study has several limitations. First, the participant pool consisted solely of young Chinese adults, limiting generalizability across ethnicities, age groups, and Fitzpatrick skin types. Second, while standardized imaging was used, in vivo application and lighting variability can affect color perception.

Finally, the potential of color cosmetics extends beyond aesthetics. As shown by Kim et al. [37], contrasting color schemes enhance concentration and alertness, as evidenced by increased β and γ wave activity, opening up interdisciplinary research between cognitive neuroscience and cosmetology.

Future studies should expand the demographic base and control for additional variables, such as skin hydration, sebum levels, and skin types. Moreover, cross‐validation with subjective user satisfaction and clinical grading could enrich model robustness. While our model demonstrates strong predictive potential, the integration of multispectral imaging or hyperspectral data may further enhance colorimetric accuracy.

## Author Contributions

G.D., Y.L., and J.R. performed the research. Y.W. designed the research study. G.D. and Y.L. contributed essential reagents or tools. G.D. and J.R. analyzed the data. G.D., Y.L., and J.R. wrote the paper. F.Y., L.L., H.M., and Y.W. reviewed and edited the draft. All authors have read and approved the final manuscript.

## Funding

The authors have nothing to report.

## Ethics Statement

The study was conducted in accordance with the Declaration of Helsinki and informed consent was obtained from all participants, and ethical approval was obtained from the Ethics Committee of Beijing Technology and Business University (reference BTBUECSR2024031, 21st February, 2024).

## Conflicts of Interest

The authors declare no conflicts of interest.

## Supporting information


**Data S1:** jocd70566‐sup‐0001‐Tables.docx.

## Data Availability

The data that support the findings of this study are available from the corresponding author upon reasonable request.
