# Supplementary material for: Applicability of Complementary Colors in Skin Tone Correction for Young Chinese Adults Based on Image Processing and Machine Learning
Source: J Cosmet Dermatol. 2025 Nov 24;24(12):e70566. doi: 10.1111/jocd.70566 (PMC12642389; doi:10.1111/jocd.70566)
Supplement: Supplementary file 1 — Data S1: jocd70566‐sup‐0001‐Tables.docx. [file JOCD-24-e70566-s001.docx]

**Supplementary materials**

**Table S1.** description analysis of facial skin tone index in different anatomical sites.

| Anatomical sites | L* | a* | b* | ITA° | Hab° |
| --- | --- | --- | --- | --- | --- |
| Forehead | 69.60±5.23 | 13.35±2.33 | 19.51±3.05 | 44.40±10.86 | 55.45±4.74 |
| Under-eye circles | 64.26±4.80 | 14.31±2.30 | 21.29±3.36 | 33.47±12.00 | 55.90±4.23 |
| Cheek | 70.43±3.92 | 14.13±2.71 | 18.07±2.64 | 48.10±8.72 | 52.04±4.40 |
| Near-nose | 68.11±4.21 | 14.08±2.96 | 20.76±3.42 | 40.87±10.19 | 55.81±5.22 |

**Table S2.** Descriptive analysis of skin tone indices (mean ± SD) across different anatomical sites pre- and post-sample application.

| Sample color | region | L* | a* | b* | ITA° | Hab° |
| --- | --- | --- | --- | --- | --- | --- |
| base | Forehead | 69.60±5.23 | 13.35±2.33 | 19.51±3.05 | 44.40±10.86 | 55.45±4.74 |
|  | Under-eye circles | 64.26±4.80 | 14.31±2.30 | 21.29±3.36 | 33.47±12.00 | 55.90±4.23 |
|  | Cheek | 70.43±3.92 | 14.13±2.71 | 18.07±2.64 | 48.10±8.72 | 52.04±4.40 |
|  | Near-nose | 68.11±4.21 | 14.08±2.96 | 20.76±3.42 | 40.87±10.19 | 55.81±5.22 |
| Green | Forehead | 71.46±4.75 | 12.14±2.51 | 17.31±2.13 | 50.47±9.00 | 55.19±4.38 |
|  | Under-eye circles | 65.08±2.53 | 12.26±2.39 | 18.20±2.50 | 39.68±7.04 | 56.23±2.88 |
|  | cheek | 71.09±3.44 | 13.10±3.86 | 16.36±2.42 | 51.90±8.15 | 52.06±5.09 |
|  | Near-nose | 69.80±4.78 | 12.80±3.83 | 17.93±3.130 | 47.21±10.91 | 55.16±4.27 |
| Purple | Forehead | 73.32±3.03 | 11.53±1.47 | 15.26±3.26 | 56.93±6.52 | 52.29±5.93 |
|  | Under-eye circles | 66.18±3.66 | 12.01±2.51 | 16.25±4.02 | 45.02±12.21 | 53.07±4.96 |
|  | cheek | 72.03±2.45 | 12.03±2.57 | 14.17±2.75 | 57.25±6.79 | 49.58±5.74 |
|  | Near-nose | 71.05±4.02 | 12.27±3.34 | 15.76±4.55 | 52.96±12.19 | 51.46±6.49 |
| White | Forehead | 71.91±4.52 | 11.75±2.25 | 16.35±2.16 | 52.64±8.86 | 54.38±4.31 |
|  | Under-eye circles | 66.68±2.10 | 12.35±2.37 | 17.82±2.63 | 43.20±7.14 | 55.36±3.21 |
|  | cheek | 72.23±2.58 | 12.20±3.03 | 16.17±2.50 | 53.92±5.76 | 53.17±6.16 |
|  | Near-nose | 71.20±4.47 | 12.31±4.15 | 18.07±3.57 | 49.13±11.13 | 56.19±5.76 |
| Blue | Forehead | 70.39±5.61 | 12.11±1.86 | 14.89±2.31 | 52.71±11.34 | 50.82±3.51 |
|  | Under-eye circles | 66.68±3.19 | 12.23±1.34 | 16.03±2.02 | 45.83±6.36 | 52.58±3.52 |
|  | cheek | 72.14±1.72 | 12.19±1.13 | 14.67±2.19 | 56.54±4.43 | 50.05±3.75 |
|  | Near-nose | 70.06±2.73 | 12.36±1.57 | 16.64±2.87 | 50.32±7.52 | 53.16±3.92 |
| Pink | Forehead | 72.55±3.38 | 11.26±2.42 | 16.02±1.25 | 54.27±6.21 | 55.19±4.00 |
|  | Under-eye circles | 68.22±2.00 | 11.85±2.25 | 17.78±1.28 | 45.62±4.58 | 56.56±3.23 |
|  | cheek | 72.65±3.75 | 12.25±2.38 | 15.03±1.19 | 56.00±6.77 | 51.07±4.56 |
|  | Near-nose | 71.16±4.54 | 11.33±2.65 | 16.41±1.64 | 51.58±9.54 | 55.72±3.56 |
| Orange | Forehead | 70.40±5.27 | 12.33±1.75 | 16.73±2.41 | 49.83±10.24 | 53.50±4.10 |
|  | Under-eye circles | 64.61±5.76 | 12.63±1.20 | 17.64±2.82 | 37.84±12.78 | 54.10±4.03 |
|  | cheek | 71.41±2.95 | 12.91±1.95 | 16.17±2.41 | 52.77±6.43 | 51.33±4.37 |
|  | Near-nose | 69.90±2.47 | 13.04±1.28 | 17.94±2.61 | 47.95±6.38 | 53.79±3.69 |
